# Synthesis and Evaluation of Fluorine-18-Labeled L-Rhamnose Derivatives

**DOI:** 10.3390/molecules28093773

**Published:** 2023-04-27

**Authors:** Xiang Zhang, Falguni Basuli, Zhen-Dan Shi, Swati Shah, Jianfeng Shi, Amelia Mitchell, Jianhao Lai, Zeping Wang, Dima A. Hammoud, Rolf E. Swenson

**Affiliations:** 1 Chemistry and Synthesis Center, National Heart, Lung, and Blood Institute, National Institutes of Health, Rockville, MD 20850, USA; 2 Center for Infectious Disease Imaging, Radiology and Imaging Sciences, Clinical Center, National Institutes of Health, Bethesda, MD 20892, USA

**Keywords:** rhamnose, fluorine-18, radiolabeling, positron emission tomography

## Abstract

The use of radiolabeled glucose for PET imaging resulted in the most commonly used tracer in the clinic, 2-deoxy-2-[^18^F]fluoroglucose (FDG). More recently, other radiolabeled sugars have been reported for various applications, including imaging tumors and infections. Therefore, in this study, we developed a series of fluorine-18-labeled L-rhamnose derivatives as potential PET tracers of various fungal and bacterial strains. Acetyl-protected triflate precursors of rhamnose were prepared and radiolabeled with fluorine-18 followed by hydrolysis to produce L-deoxy [^18^F]fluororhamnose. The overall radiochemical yield was 7–27% in a 90 min synthesis time with a radiochemical purity of 95%. In vivo biodistribution of the ligands using PET imaging showed that 2-deoxy-2-[^18^F]fluoro-L-rhamnose is stable for at least up to 60 min in mice and eliminated via renal clearance. The tracer also exhibited minimal tissue or skeletal uptake in healthy mice resulting in a low background signal.

## 1. Introduction

Structural imaging along with microbiological confirmation have long been the mainstay of the management of infectious diseases. However, structural imaging is nonspecific and in cases with deep seated infections, invasive methods such as biopsies or bronchoalveolar lavage are needed to provide microbiological analysis, sometimes with inconclusive results [1,2]. Positron emission tomography (PET) is a non-invasive imaging technique that measures the body’s biochemical functions and metabolism. When coupled with CT or MRI, PET has been widely acknowledged in detecting tumors, infections, and systemic inflammatory diseases in different stages [3,4,5,6]. Fluorine-18 radiolabeled glucose, 2-deoxy-2-[^18^F]fluoroglucose (FDG), is the most commonly used ligand in the clinic, mainly in cancer imaging. It has recently been approved for the imaging of infection and inflammation [7]. FDG is, however, unable to differentiate infectious etiologies from other causes of increased glucose metabolism, such as tumors and sterile inflammation.

**Figure 1:**
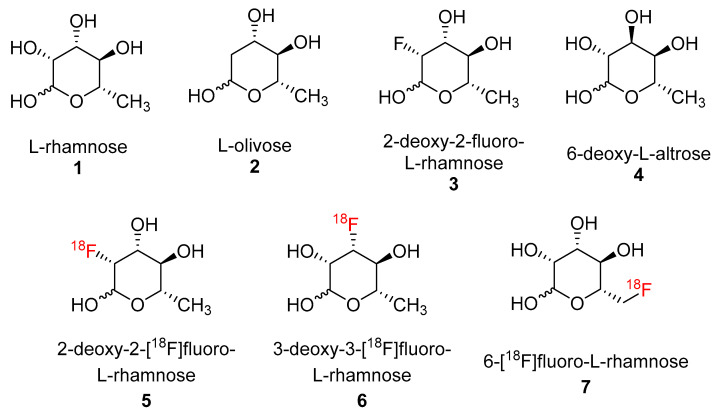
Structure of L-rhamnose (**1**) and derivatives (**2**–**4**) [22]; fluorine-18-labeled L-rhamnose analogues proposed for PET imaging of infections (**5**–**7**, this work).

More recently, other radiolabeled sugars have been developed for different purposes, such as imaging tumors [8] but more commonly for imaging different infections [9,10,11]. L-Rhamnose is a naturally occurring 6-deoxy sugar that is commercially used in the food, beverage, cosmetic and pharmaceutical industries (Figure 1, compound **1**) [12,13], but has no role in mammalian metabolism [14]. In *E. coli*, it is catabolized by a pathway involved with isomerization and phosphorylation [15], and in multiple fungal species, it is degraded through non-phosphorylated, aldolase glycolysis [16,17,18,19,20]. In addition, exclusively bacterial D- and L-sugars, including L-Rhamnose, are known to be incorporated into higher-order bacterial glycans [21].

In 2016, Liu et al. reported a series of L-rhamnose derivatives with optimized metabolic profiles (Figure 1, compounds **2**–**4**) [22]. Their work has provided a platform for the development of radiolabeled L-rhamnose derivatives which might have potential for distinguishing infections from other pathologies and possibly distinguishing different pathogens (e.g., fungi vs. bacteria). Herein, we report our results in developing a series of L-rhamnose-based PET imaging probes (Figure 1, compounds **5**–**7**).

## 2. Results and Discussion

### 2.1. Chemistry

**Scheme 1:**
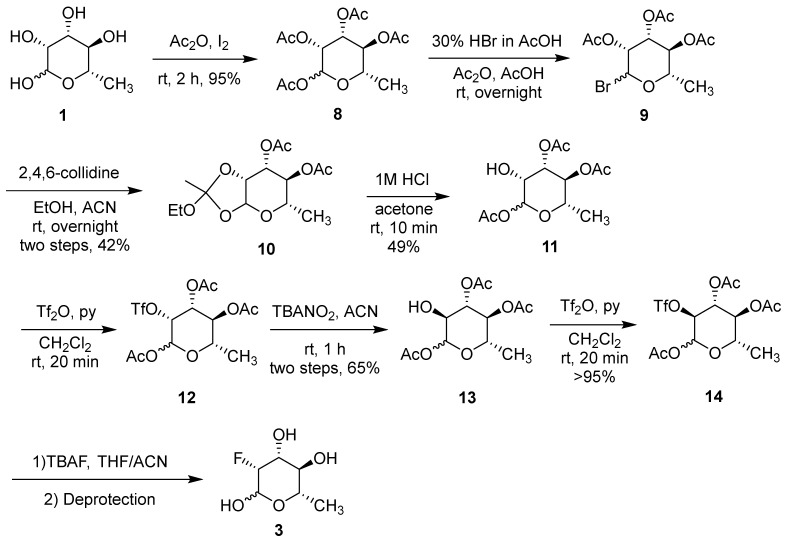
Synthesis of 2−deoxy−2−fluoro−L−Rhamnose (**3**) and the triflate precursor **14**.

Compound **3** reported by Liu et al. provided a base for this PET probe developmental work [22]. The fluorine on the 2-position of Compound **3** provided a suitable labeling site for fluorine-18, the most commonly used positron-emitting isotope for PET imaging studies. Inspired by the tracer-development work of FDG derivatives [23,24,25], the 3 and 6-fluoro analogues of rhamnose were designed to compare their in vivo properties. The radiosynthesis of **5** was accomplished via nucleophilic substitution from the trifluoromethanesulfonate (triflate) precursor 14 followed by hydrolysis, similar to the synthesis of FDG [26]. The synthesis of the triflate precursor was achieved following a procedure found in the literature, with minor modifications (Scheme 1) [27]. Briefly, commercially available L-rhamnose (**1**) was used as the starting material. Per-*O*-acetylation of **1** through the iodine-promoted acetylation [28] resulted in a near-quantitative formation of pentaacetate **8**. A two-steps selective O-deacetylation on the 2-position was performed according to the literature procedure [27]. The key steps to produce ortho−ester **10** were achieved via 1-bromination of compound **8** to produce compound **9**, followed by a reaction with EtOH and 2,4,6-collidine. The bromo derivative **9** is unstable; therefore, the reaction to prepare **10** was performed immediately with the freshly prepared **9**. The subsequent hydrolysis in HCl/acetone yielded the 2-hydroxyl derivative **11**. To achieve the axial 2-fluorine in the desired product **5** through a nucleophilic substitution (S_N_2) reaction, an equatorial triflate is necessary for the precursor **14**. Therefore, a stereo inversion of the 2-hydroxyl group of compound **11** was performed via a two-steps Lattrell–Dax epimerization reaction [29]. Compound **11** was converted to triflate derivative **12** first, followed by a nitrite-mediated epimerization. The equatorial 2-hydroxyl epimer **13** was obtained in a reasonably good yield (65%). The desired triflate precursor **14** was synthesized quantitatively from compound **13**. Synthesis of the reference non-radioactive standard, 2-deoxy-2-fluoro-L-Rhamnose (**3)** was initially attempted with the typical DAST fluorination of **13**; however, no desired product was observed, probably due to the low stability of **13** under the reaction condition. With the milder TBAF fluorination condition mimicking the radiofluorination, **3** was obtained from its triflate precursor **14** in a 6% yield.

**Scheme 2:**
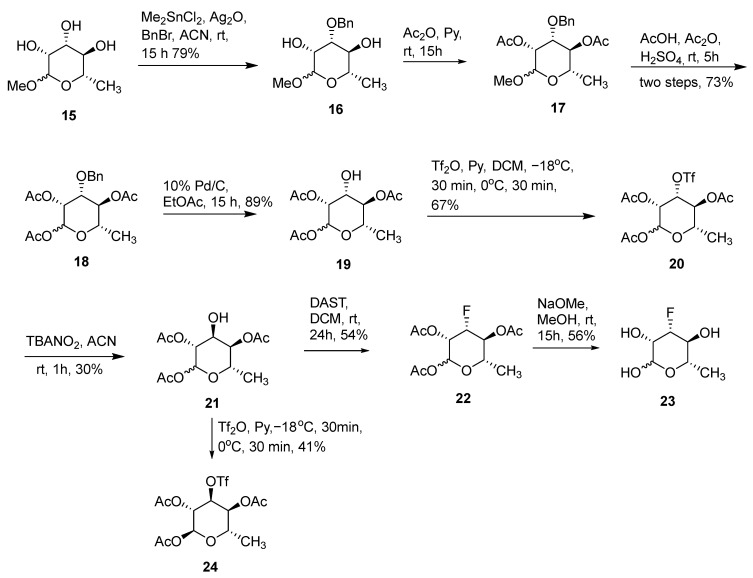
Synthesis of 3-deoxy-3-fluoro-L-Rhamnose (**23**) and the triflate precursor **24**.

The synthesis of non−radioactive standard, 3-deoxy-3-fluoro-L-Rhamnose (**23**), and the radiolabeling precursor **24** were started from commercially available methyl-L-rhamnopyranoside **15** (Scheme 2). First, the 3-hydroxyl group was selectively protected to achieve benzyl derivative **16** using a dimethyltin dichloride catalyzed reaction. Acetylation on the rest of the hydroxyl groups was performed to obtain **17**. Without purification, the 1-methoxy group on crude compound **17** was converted to acetate to produce **18** in a good yield over two-steps. Subsequent de-benzylation with palladium on carbon produced 3-hydroxyl compound **19**. A similar Lattrell–Dax epimerization as described above for the synthesis of compound **13** was performed to obtain the stereo-inverted epimer **21**. Fluorination with DAST, followed by de-acetylation, successfully produced **23** in a moderate yield. The triflate precursor **24** was synthesized by treating **21** with triflic anhydride/pyridine using a standard condition.

**Scheme 3:**
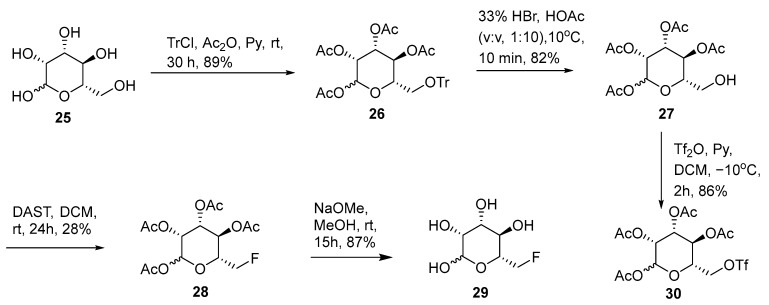
Synthesis of 6-fluoro-L-Rhamnose (**29**) and the triflate precursor **30**.

Synthesis of non−radioactive standard, 6-fluoro-L-Rhamnose (**29**), and the triflate precursor **30** (Scheme 3) was more straight-forward. Starting from commercially available L-mannose **25**, selective protection of the primary hydroxyl group on 6-CH_2_OH was achieved using triphenylmethyl chloride, followed by a per-O-acetylation to provide **26**. The triphenylmethyl protective group was then removed to obtain **27**. The non-radioactive standard **29**, was obtained from the reaction of **27** with DAST, followed by de-acetylation under the same condition as above. Similarly, the triflate precursor **30** was synthesized by treating the hydroxyl derivative **27** with triflic anhydride/pyridine.

### 2.2. Radiochemistry

**Scheme 4:**
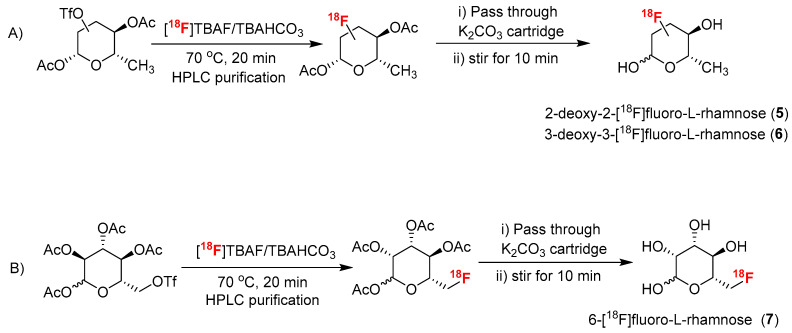
Syntheses of fluorine-18-labeled rhamnose derivatives. (**A**): **5**, **6**; (**B**): **7**.

The radiosyntheses of the target probes **5**–**7** were achieved in two-steps: fluorination and the deprotection of the acetyl-protecting group (Scheme 4). Labeling efficiency was first standardized manually using 3–10 mCi of [^18^F]fluoride. In general, the cyclotron-produced [^18^F]fluoride was trapped on an anion exchange cartridge, and was eluted with a water/methanolic solution of tetrabutylammonium bicarbonate (TBAB) [30]. TBAB was selected because it was reported to be more efficient for the radiofluorination of base-sensitive precursors. After azeotropic drying with anhydrous acetonitrile, the precursor solution was added and the radiofluorination was performed at 70 °C. The progress of the reaction was monitored using analytical high-performance liquid chromatography (HPLC). The fluorine-18-labeled intermediate was purified via HPLC using a semi-preparative column.

**Figure 2:**
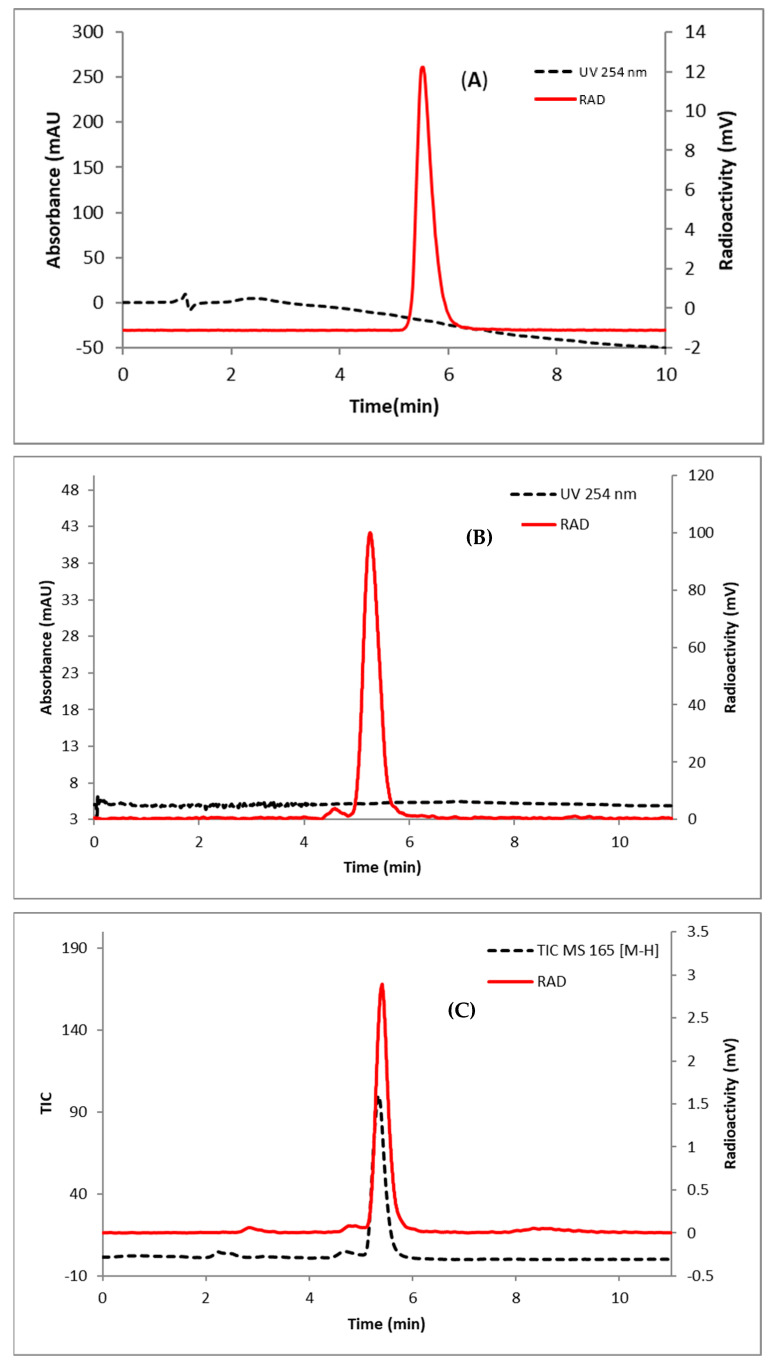
A representative HPLC chromatogram of (**A**) Fluorine-18-labeled intermediate of compound **6**; (**B**) Compound **6**; (**C**) LCMS of compound **6** with the co-injected non-radioactive standard; HPLC conditions: (**A**) Phenomenex Luna column (100 × 4.6 mm), 3.5 µm. Using 5–95% organic phase in 8 min; flow rate 1 mL/min. (**B**,**C**) Waters BEH Amide column (150 × 4.6 mm), 3.5 µm. Using 90–50% organic phase in 16 min; flow rate 0.5 mL/min. LC-MS/MS ion analysis for m/s 165 [M-H]^−^. Aqueous phase: 95% water + 5% ACN with 0.1% NH_4_OH; organic phase: 95% ACN + 5% water with 0.1% NH_4_OH.

**Figure 3:**
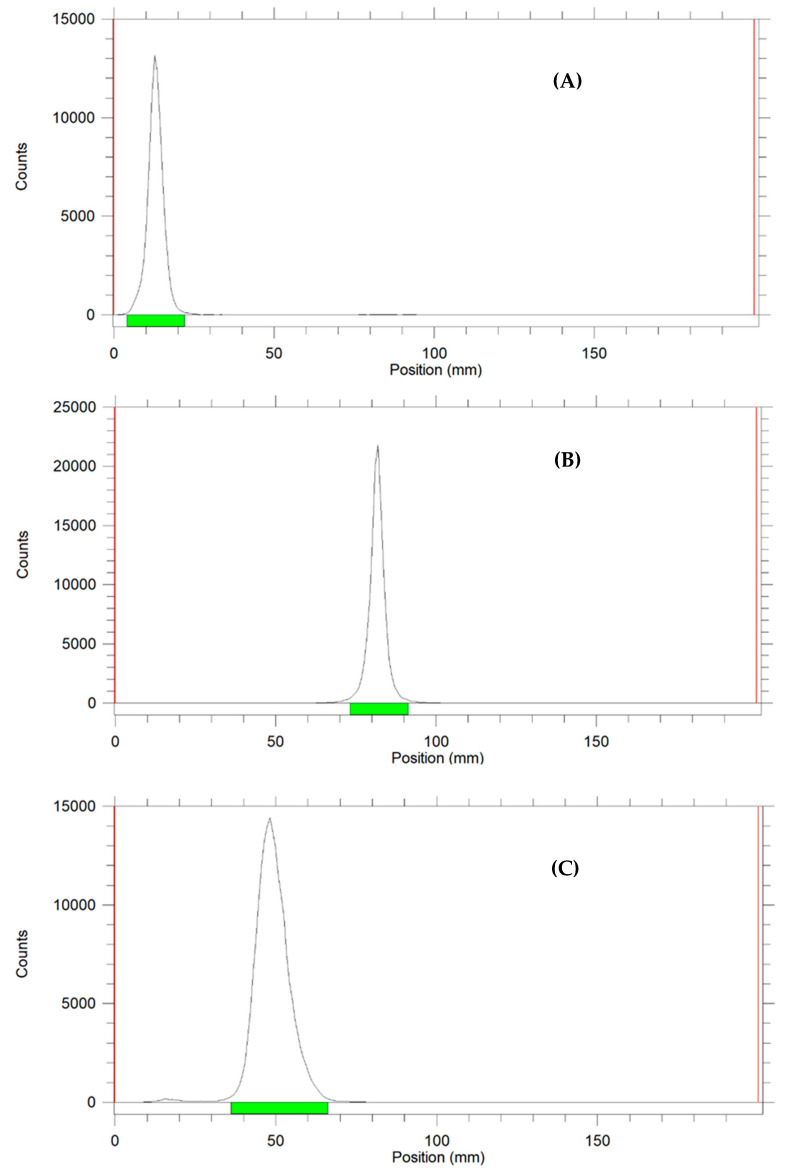
Radio-TLC chromatogram of (**A**) [^18^F]fluoride; (**B**) intermediate compound **6**; (**C**) compound **6**. TLC developing solvent: 25% methanol in dichloromethane.

The collected fraction was diluted with water and trapped on a solid phase extraction (SPE) cartridge. The trapped intermediate was eluted with ethanol through a potassium carbonate cartridge into vial containing a stir bar. The eluted mixture was stirred for 10 min and neutralized. The final product was retained on an FDG purification cartridge and eluted with normal saline. The radiochemical yields (RCYs) for **5**–**7** were 7–12%, 10–15%, and 18–25%, respectively (uncorrected, *n* > 3), in ~90 min. Radiochemical purities were >95%. The identity of the final radiolabeled product was confirmed via liquid chromatography mass spectrometry (LCMS) by co-eluting with a non-radioactive standard. A representative HPLC for compound **6** is presented in Figure 2. A radio TLC analysis revealed the absence of [^18^F]fluoride in the labeled intermediate and the final product (Figure 3).

**Figure 4:**
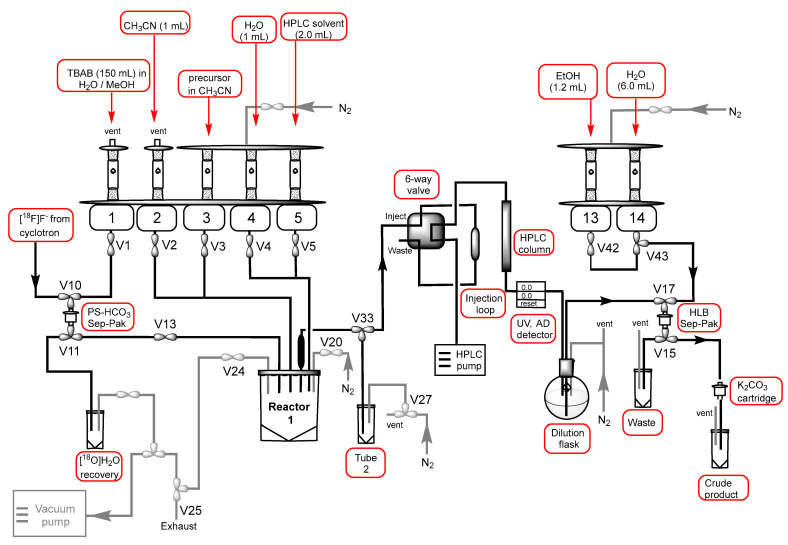
Schematic diagram of the automated synthesis of in GE FX-N Pro synthesizer.

After successful manual standardization of the procedure, an automated synthesis was performed in a GE Tracerlab FX-N module (Figure 4). The synthesis consisted of seven reagent vials. Vials 1–5 were used for the elution of [^18^F]fluoride, drying, and fluorination reaction. Vials 13 and 14 were used for the trap and release of the purified intermediate. The valve 15 normal close was connected to the crude product vial through an inline K_2_CO_3_ cartridge for hydrolysis. The crude product was formulated by trapping on an FDG cartridge and eluting with normal saline.

All three tracers were stable in saline (pH 6) at room temperature for at least 4 h. Compound **5** was stable at 37 °C in whole human serum and compound **7** was slowly decomposed (Table 1). However, compound **6** rapidly decomposed resulting in 70% being intact after 4 h (Figure S1).

### 2.3. PET/CT Imaging

**Figure 5:**
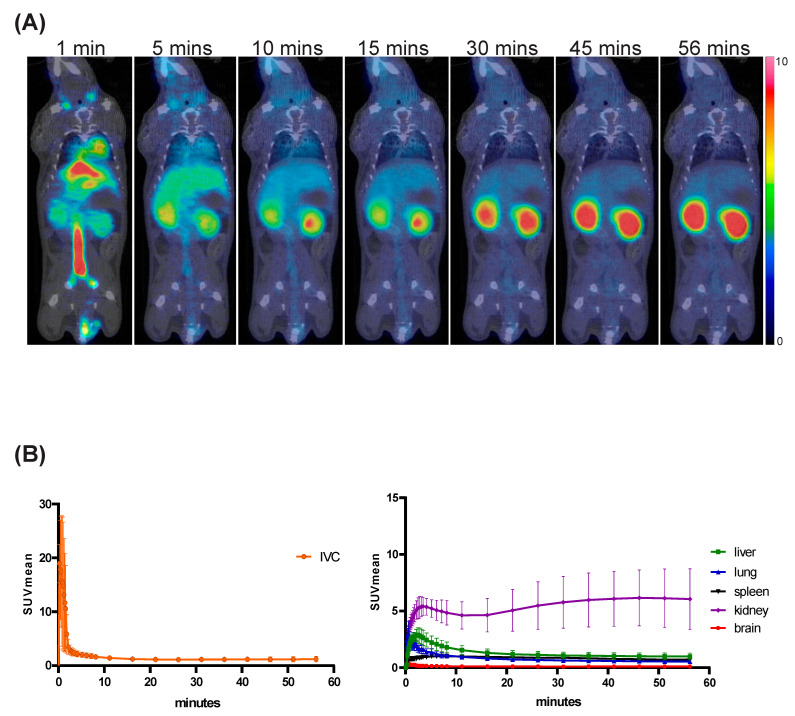
Dynamic PET/CT imaging of 2-deoxy-2-[^18^F]-fluoro-L-rhamnose in control mice. A total of 60 min of dynamic PET imaging was performed. (**A**) Representative coronal sections of PET/CT scan images with frames from 1, 5, 10, 15, 30, 45, and 56 min are shown. (**B**) Average time–activity curves (n = 4) from various organs.

**Figure 6:**
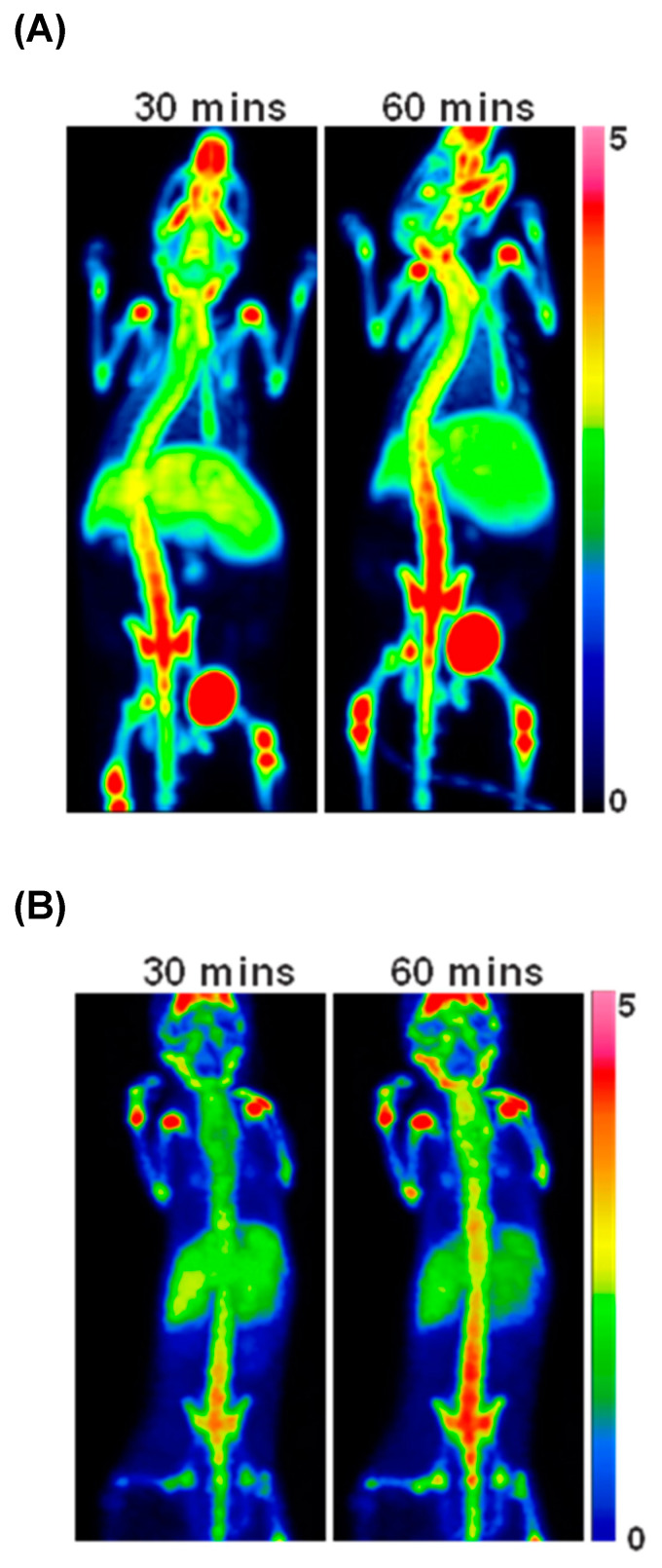
PET/CT imaging with 6-[^18^F]-fluoro-L-rhamnose (**A**) and 3-deoxy-3-[^18^F]fluoro-L-rhamnose (**B**). Representative PET/CT images of control mice demonstrate skeletal uptake, suggesting rapid defluorination of the ligand.

Representative dynamic PET images for a healthy mouse injected with a single bolus of 2-deoxy-2-[^18^F]fluoro-L-rhamnose (**5**) and the averaged time activity curves are shown in Figure 5A,B. The images indicate that after the initial blood pool clearance, the tracer shows little to no tissue uptake in the lungs, heart, liver, spleen, or brain and is quickly eliminated by renal clearance. These results suggest that **5** is not metabolized by mammalian cells, as expected. Additionally, it was not detected within the skeletal system at any point and thus the ligand is stable for at least 60 min in vivo. The time activity curves showed that **5** has a short biological half-life of 3.4 ± 1.7 min. The in vivo biodistribution of the other two radiolabeled derivatives of rhamnose (**6** and **7**) were also obtained using dynamic PET imaging. Both **6** and **7** showed rapid defluorination upon injection in mice, as evidenced by the increasing skeletal uptake in the representative PET images (Figure 6).

## 3. Material and Methods

### 3.1. General

Methyl-rhamnopyranoside was obtained from Combi-Blocks Inc. (San Diego, CA, USA). All other chemicals and solvents were received from Sigma Aldrich (St. Louis, MO, USA) and used without further purification. Fluorine-18 was received from the National Institutes of Health cyclotron facility (Bethesda, MD, USA). A Chromafix PS-HCO3 cartridge was purchased from Synthra GmbH (Hamburg Germany). Oasis HLB plus and K_2_CO_3_ cartridges were obtained from Waters (Milford, MA, USA). An FDG cartridge was purchased from NucMedCor (San Francisco, CA, USA). Mass spectrometry (MS) was performed on a 6130 Quadrupole LC/MS, Agilent Technologies instrument equipped with a diode array detector. ^1^H, ^13^C, and ^19^F NMR spectra were recorded on a Varian spectrometer (400 MHz). Chemical shifts (ppm) were reported relative to the solvent residual peaks. HPLC for purification and analytical analysis were performed on an Agilent 1200 Series instrument equipped with multi-wavelength detectors along with a flow count radiodetector (Eckert & Ziegler, B-FC-3500 diode). LC-MS/MS analysis was performed on an Agilent 6460C triple quadrupole mass spectrometer with an ESI source and a flow count radiodetector (Eckert & Ziegler, B-FC-3500 diode). The LC unit was an Agilent 1200 series chromatographic system equipped with a 1260 binary pump, 1290 thermostatted column compartment and 1260 high-performance autosampler. Instrument control and data processing were performed using Aglient’s MassHunter Software.

### 3.2. Chemical Syntheses

*(3R,4R,5S,6S)-6-methyltetrahydro-2H-pyran-2,3,4,5-tetrayl tetraacetate* **(8)**. The synthetic process followed the literature [27]. *L*-Rhamnose (**1**, 10 g, 54.9 mmol) was carefully added in portions (3 portions in 15 min) to a stirring solution of iodine (0.125 g, 0.49 mmol) in acetic anhydride (60 mL) in a cool-water bath (10–15 °C). The resulting mixture was allowed to warm up to room temperature and stirred for 2 h. The mixture was then poured onto a mixture of crushed ice and saturated aqueous Na_2_S_2_O_3_ (250 mL, 1:1 mixture) with vigorous stirring. To the resulting light-yellow mixture in an ice-water bath, NaHCO_3_ was added portion wise until no more CO_2_ was released. The crude product was extracted with CH_2_Cl_2_ (150 mL × 3). The organic layer was combined, washed with saturated NaHCO_3_ solution and water (400 mL each), and dried over anhydrous Na_2_SO_4_. Crude product **8** was obtained by removing the volatiles under reduced pressure (19.33 g, 95.4% yield, α:β anomer ratio = 3:1). ^1^H NMR (400 MHz, Chloroform-*d*) δ 6.02 (d, *J* = 1.9 Hz, 1H), 5.83 (s, 0.34H), 5.48 (s, 0.34H), 5.36–5.28 (m, 1H), 5.26 (dd, *J* = 3.5, 2.0 Hz, 1H), 5.18–5.05 (m, 1.7H), 4.00–3.90 (m, 1H), 3.72–3.62 (m, 0.36H), 2.23 (s, 4H), 2.22, (s, 1H), 2.20 (s, 3H), 2.15 (s, 3H), 2.11 (s, 1H), 2.07 (s, 4H), 2.01 (s, 4H), 1.30 (d, *J* = 6.2 Hz, 1H), 1.25 (d, *J* = 6.2 Hz, 3H). MS (ESI) calculated mass for the parent C_14_H_20_O_9_ 332.11 [M], found 355.00 [M + Na].

*(3R,4R,5S,6S)-2-bromo-6-methyltetrahydro-2H-pyran-3,4,5-triyl triacetate* **(9).** To a solution of compound **8** (19.33 g, 58.2 mmol) in glacial acetic acid (20 mL) and acetic anhydride (1.6 mL), HBr in acetic acid (30%, 20 mL) was added dropwise in an ice-water bath and vigorous stirring. The resulting mixture was stirred at rt overnight and slowly quenched with a pre-cooled saturated NaHCO_3_ solution (500 mL). The brominated intermediate was extracted with CHCl_3_ (200 mL × 2). The organic layer was combined and dried over anhydrous Na_2_SO_4_. The bromide intermediate **9** was obtained by removing the volatiles under reduced pressure as a yellow oil (17.8 g).

*(5S,6S,7R,7aR)-2-ethoxy-2,5-dimethyltetrahydro-5H-[1,3]dioxolo [4,5-b]pyran-6,7-diyl diacetate* **(10).** The oily bromide intermediate **9** was dissolved in a mixture of anhydrous acetonitrile (8 mL), and 2,4,6-collidine (11 mL) and ethanol (200 proof, 13 mL) was added. The resulting mixture was stirred at rt overnight, diluted with CH_2_Cl_2_ (300 mL), and washed with water (300 mL × 2) and brine (200 mL). The organic layer was dried over anhydrous Na_2_SO_4_. Crude product was obtained by removing the volatiles under reduced pressure. Product **10** was purified using flash column chromatography with hexane/ethyl acetate 4/1 to 2/1 gradient (7.8 g, 42.2% yield for 2 steps). ^1^H NMR (400 MHz, Chloroform-*d*) δ 5.41 (d, *J* = 2.4 Hz, 1H), 5.16–5.02 (m, 2H), 4.59 (dd, *J* = 3.8, 2.4 Hz, 1H), 3.65–3.47 (m, 3H), 2.12 (s, 3H), 2.07 (s, 3H), 1.75 (s, 3H), 1.30–1.14 (m, 6H). MS (ESI) calculated mass for the parent C_14_H_22_O_8_ 318.13 [M], found 341.00 [M + Na].

*(3R,4S,5S,6S)-3-hydroxy-6-methyltetrahydro-2H-pyran-2,4,5-triyl triacetate* **(11).** Hydrochloric acid (1 N, 10 mL) was added to a solution of the orthoester **10** (7 g, 22.0 mmol) and acetone (15 mL). The mixture was stirred at rt for 10 min and volatiles were removed under reduced pressure. The resulting crude product was dissolved in CH_2_Cl_2_ (150 mL) and washed with water (150 mL × 2). The organic layer was dried over anhydrous Na_2_SO_4_. Crude product was obtained by removing the volatiles under reduced pressure. Product **11** was purified using flash column chromatography with hexane/ethyl acetate 4/1 to 1/1 gradient (3.15 g, 49.3% yield). ^1^H NMR (400 MHz, Chloroform-*d*) δ 5.76 (s, 1H), 5.15 (t, *J* = 9.8 Hz, 1H), 4.99 (dd, *J* = 9.9, 3.0 Hz, 1H), 4.22–4.15 (m, 1H), 3.65 (dq, *J* = 9.3, 6.2 Hz, 1H), 2.5–2.25 (br s, 1H), 2.17 (s, 3H), 2.11 (s, 3H), 2.06 (s, 3H), 1.27 (d, *J* = 6.2 Hz, 3H). MS (ESI) calculated mass for the parent C_12_H_18_O_8_ 290.10 [M], found 313.00 [M + Na].

*(3S,4S,5S,6S)-3-hydroxy-6-methyltetrahydro-2H-pyran-2,4,5-triyl triacetate* **(13).** The triacetate **11** (1.0 g, 3.19 mmol) was dissolved in anhydrous CH_2_Cl_2_ (20 mL) and anhydrous pyridine (3.5 mL) and cooled with an ice-salt bath. Trifluoromethanesulfonic anhydride (4.5 g, 15.97 mmol) in CH_2_Cl_2_ (10 mL) was added dropwise. The mixture was stirred at rt for 20 min, and then sequentially washed with HCl (0.3 M, 30 mL), saturated NaHCO_3_ (30 mL), and brine (30 mL). The organic layer was dried over anhydrous Na_2_SO_4_. The crude triflate was obtained by removing the volatiles under reduced pressure.

The crude triflate (**12**, 1.35 g) was stirred with acetonitrile (30 mL) and tetrabutylammonium nitrate (4.59 g, 16.0 mmol) at rt for 1 h. Crude product was obtained by removing the volatiles under reduced pressure. Product **13** was purified using flash column chromatography with hexane/ethyl acetate 3/1 to 1/1 gradient (0.65 g, 65% yield for 2 steps). ^1^H NMR (400 MHz, Chloroform-*d*) δ 5.58 (d, *J* = 8.3 Hz, 1H), 5.07 (t, *J* = 9.5 Hz, 1H), 4.77 (t, *J* = 9.6 Hz, 1H), 3.76–3.60 (m, 2H), 3.05 (s, 1H), 2.15 (s, 3H), 2.07 (s, 3H), 2.04 (s, 3H), 1.20 (d, *J* = 6.2 Hz, 3H). MS (ESI) calculated mass for the parent C_12_H_18_O_8_ 290.10 [M], found 313.00 [M + Na].

*(3S,4R,5S,6S)-6-methyl-3-(((trifluoromethyl)sulfonyl)oxy)tetrahydro-2H-pyran-2,4,5-triyl triacetate* **(14).** The triacetate **13** (0.52 g, 1.79 mmol) was dissolved in anhydrous CH_2_Cl_2_ (20 mL) and anhydrous pyridine (1.2 mL) and cooled with an ice-salt bath. Trifluoromethanesulfonic anhydride (1.52 g, 5.37 mmol) in CH_2_Cl_2_ (10 mL) was added dropwise. The mixture was stirred at rt for 20 min, and then sequentially washed with HCl (0.3 M, 30 mL), saturated NaHCO_3_ (30 mL), and brine (30 mL). The organic layer was dried over anhydrous Na_2_SO_4_. Crude product was obtained by removing the volatiles under reduced pressure. Flash column chromatography was used to purify product **14** with hexane/ethyl acetate 3/1 to 1/1 gradient (0.75 g, quant. yield). ^1^H NMR (400 MHz, Chloroform-*d*) δ 5.80 (d, *J* = 8.3 Hz, 1H), 5.38 (t, *J* = 9.6 Hz, 1H), 4.89–4.76 (m, 2H), 3.78 (dq, *J* = 9.7, 6.1 Hz, 1H), 2.16 (s, 3H), 2.07 (s, 3H), 2.05 (s, 3H), 1.25 (d, *J* = 6.2 Hz, 3H). ^13^C NMR (101 MHz, CDCl_3_) δ 169.53, 169.51, 168.44, 118.21 (q, *J* = 319.0 Hz), 90.21, 80.92, 77.35, 77.23, 77.03, 76.71, 73.02, 71.27, 71.24, 20.49, 20.37, 20.26, 17.02. MS (ESI) calculated mass for the parent C_13_H_17_F_3_O_10_S 422.05 [M], found 362.90 [M-OAc].

*(3R,4R,5R,6S)-3-fluoro-6-methyltetrahydro-2H-pyran-2,4,5-triol (2-deoxy-2-fluoro-L-rhamnose*, **(3).** To a solution of 2-hydroxyl analogue **14** (50 mg, 0.118 mmol) in anhydrous acetonitrile (2 mL), TBAF in THF (1.0 M, 0.177 mL, 0.177 mmol) was added. The solution was stirred at 65 °C overnight. The volatiles were removed under reduced pressure. Flash column chromatography was used to purify product **7** with hexane/ethyl acetate 5/1 to 2/1 gradient (3.5 mg, 10% yield, α:β anomer ratio = 1:1). ^1^H NMR (400 MHz, Chloroform-*d*) δ 6.02 (s, 1H), 5.78 (d, *J* = 60 Hz, 1H), 5.35–5.30 (m, 1 H), 3.30–5.25 (m, 1H), 5.18–5.10 (m, 2H), 5.08–4.95 (m, 1H), 4.88 (dd, *J* = 120, 4.0 Hz, 1H), 4.00–3.80 (m, 1H), 3.73–3.65 (m, 1H), 2.20 (s, 3H), 2.09 (s, 3H), 2.08 (s, 3H), 2.12 (s, 3H), 2.08 (s, 3H), 2.02 (s, 3H), 1.30 (d, *J* = 6.0 Hz, 3H), 1.25 (d, *J* = 6.0 Hz, 3H). MS (ESI) calculated mass for the parent C_12_H_17_FO_7_ 292.10 [M], found 273.10 [M-F]. Triacetate (3.5 mg, 0.012 mmol) was dissolved in TFA (1.0 mL) and stirred at 50 °C for 1 h. The volatiles were removed under a reduced pressure to yield 2-deoxy-2-fluoro-L-rhamnose (**25**) as a yellow oil (1.2 mg, 6%). The ^19^F NMR chromatogram was compared with the literature which found an identical result [22].

*(3R,4R,5S,6S)-4-(benzyloxy)-2-methoxy-6-methyltetrahydro-2H-pyran-3,5-diol* **(16).** Methyl-rhamnopyranoside **15** (2.85 g, 16.0 mmol), benzyl bromide (2.91 mL, 24 mmol), dimethyltin dichloride (351 mg, 1.6 mmol), and Ag_2_O (4.07 g, 17.6 mmol) were stirred in anhydrous acetonitrile (90 mL) at room temperature for 15 h. After being filtered through a celite pad, the filtrate was evaporated and the residue was purified using silica gel flash chromatography to afford **16** as a colorless oil (3.41 g, 79%, α:β = 1). β-isomer: ^1^H NMR (400 MHz, CDCl_3_) δ 7.30–7.32 (m, 5H), 4.71 (d, 1H, *J* = 1.6 Hz), 4.70 (d, 1H, *J* = 11.3 Hz), 4.57 (d, 1H, *J* = 11.3 Hz), 4.02 (dd, 1H, *J* = 1.6 and 3.1 Hz), 3.67–3.61 (m, 2H), 3.56 (m, 1H), 3.36 (s, 3H), 1.32 (d, *J* = 6.3 Hz, 3H). α-isomer: ^1^H NMR (400 MHz, CDCl_3_) δ 7.39–7.32 (m, 5H), 4.75 (d, 1H, *J* = 11.3 Hz), 4.74 (s, 1H), 4.52 (d, 1H, *J* = 11.7 Hz), 3.72–3.68 (m, 2H), 3.60 (m, 1H), 3.42 (t, 1H, *J* = 9.0 Hz), 3.35 (s, 3H), 1.34 (d, *J* = 6.3 Hz, 3H). MS (ESI) calculated mass for the parent C_14_H_20_O_5_ 268 [M], found 268 [M].

*(3R,4R,5S,6S)-4-(benzyloxy)-5-hydroxy-6-methyltetrahydro-2H-pyran-2,3-diyldiacetate* **(18).** Compound **16** (3.24 g, 12.1 mmol) was dissolved in anhydrous pyridine (12 mL) and Ac_2_O (7 mL). The solution was stirred at room temperature for 15 h. Solvents were evaporated and the residue was dissolved in EtOAc (300 mL), washed with sat. NaHCO_3_, 1 N HCl, H_2_O, and brine, and dried over Na_2_SO_4_. After the evaporation of solvents, the crude product **17** was used for next step. H_2_SO_4_ (0.6 mL) was added dropwise to a solution of **17** (4.25 g, 12.1 mmol) in Ac_2_O (20 mL) and the solution was stirred at room temperature for 5 h. The reaction mixture was poured into a stirred mixture of ethyl acetate (150 mL) and sat. NaHCO_3_ (80 mL). The organic phase was separated and washed with sat. NaHCO_3_ and brine and dried over Na_2_SO_4_. After the evaporation of solvents, the residue was purified using silica gel flash chromatography to afford the product **18** as a colorless oil (3.37 g, 73%). ^1^H NMR (400 MHz, CDCl_3_): δ 7.37–7.26 (m, 5H), 6.12 (d, 0.27H, *J* = 2.0 Hz), 6.03 (d, 0.73H, *J* = 2.0 Hz), 5.34 (dd, 0.73H, *J* = 2.0 and 3.5 Hz), 5.23 (m, 0.27H), 5.16 (m, 0.27H), 5.07 (t, 0.73H, *J* = 9.0 Hz), 4.72–4.43 (m, 2H), 3.94–3.79 (m, 2H), 2.16 (s, 2.19H), 2.12 (s, 0.81H), 2.11 (s, 2.19H), 2.10 (s, 0.81H), 2.05 (s, 0.81H), 2.04 (s, 2.19H), 1.23 (d, *J* = 6.3 Hz, 0.81H), 1.21(d, *J* = 6.3 Hz, 2.19H). MS (ESI) calculated mass for the parent C_19_H_24_O_8_ 380 [M], found 403 [M + Na].

*(3R,4R,5R,6S)-4-hydroxy-6-methyltetrahydro-2H-pyran-2,3,5-triyl triacetate* **(19).** 10% Pd/C (1.5 g) was added to **18** (3.15 g, 8.28 mmol) in EtOAc (200 mL). The mixture was stirred at room temperature under a H_2_ atmosphere for 2 h and filtered through a celite pad. The filtrate was evaporated and the residue was purified using silica gel flash chromatography to afford **19** as a white solid (2.14 g, 89%). ^1^H NMR (400 MHz, CDCl_3_): δ 6.10 (d, 0.26H, *J* = 2.0 Hz), 6.06 (d, 0.74H, *J* = 1.6 Hz), 5.25 (dd, 0.26H, *J* = 3.1 and 9.8 Hz), 5.17 (m, 0.26H), 5.09 (dd, 0.74H, *J* = 1.8 and 13.7 Hz), 4.90 (t, 0.74H, *J* = 9.8 Hz), 4.10–4.00 (m, 1H), 3.97–3.84 (m, 1H), 2.16 (s, 2.22H), 2.12 (s, 0.78H), 2.11 (s, 2.22H), 2.10 (s, 0.78H), 2.05 (s, 0.78H), 2.04 (s, 2.22H), 1.23 (d, *J* = 6.3 Hz, 0.78H), 1.21(d, *J* = 6.3 Hz, 2.22H). MS (ESI) calculated mass for the parent C_12_H_18_O_8_ 290 [M], found 313 [M + Na].

*(3R,4R,5S,6S)-6-methyl-4-(((trifluoromethyl)sulfonyl)oxy)tetrahydro-2H-pyran-2,3,5-triyl triacetate* **(20).** Trifluoromethanesulfonic anhydride (0.33 mL, 1.94 mmol) was added to a mixture of compound **19** (508 mg, 1.75 mmol) and pyridine (0.22 mL) in dichloromethane (18 mL) at −18 °C. After stirring for 0.5 h, the mixture was warmed up to 0 °C and stirred for an additional 0.5 h. Water (50 mL) was added and the organic layer was separated. The aqueous layer was extracted with dichloromethane (3 × 50 mL). The combined organic layers were washed with 10% H_2_SO_4_, sat. NaHCO_3_, and brine and dried over MgSO_4_. After the evaporation of solvents, the residue was purified using silica gel flash chromatography to afford product **20** as a colorless oil (494 mg, 67%). ^1^H NMR (400 MHz, CDCl_3_): δ 6.06 (d, 1H, *J* = 2.0 Hz), 5.38 (dd, 1H, *J* = 2.0 and 3.5 Hz), 5.28 (t, 1H, *J* = 9.8 Hz), 5.18 (dd, 1H, *J* = 3.7 and 10.0 Hz), 3.92 (m, 1H), 2.21 (s, 3H), 2.18 (s, 3H), 2.15 (s, 3H), 1.27 (d, *J* = 6.3 Hz, 3H). ^19^F NMR (376 MHz, CDCl_3_): δ −75.0. MS (ESI) calculated mass for the parent C_13_H_17_F_3_O_10_S 422 [M], found 445 [M + Na].

*(3R,4S,5R,6S)-4-hydroxy-6-methyltetrahydro-2H-pyran-2,3,5-triyl triacetate* **(21).** Compound **20** (422 mg, 1.0 mmol) was dissolved in dry CH_3_CN (2 mL) and solid tetrabutylammonium nitrite (1.44 g, 5 mmol) was added. After stirring for 1 h at rt, the reaction mixture was evaporated. The residue was dissolved in CH_2_Cl_2_, washed with brine, and dried over MgSO_4_. After the evaporation of solvents, the residue was purified using silica gel flash chromatography to afford product **21** as a white solid (87 mg, 30%). ^1^H NMR (400 MHz, CDCl_3_): δ 5.95 (d, 0.84H, *J* = 2.3 Hz), 5.91 (s, 0.16H), 5.10 (m, 0.16H), 5.02 (dd, 0.16H, *J* = 1.6 and 3.5 Hz), 5.00 (dd, 0.84H, *J* = 2.3 and 4.3 Hz), 4.89 (dd, 0.84H, *J* = 3.3 and 8.8 Hz), 4.27 (m, 0.84H), 4.12–4.06 (m, 1H), 3.74 (m, 0.16H), 2.15 (s, 0.48H), 2.13 (s, 0.84 × 6H), 2.12 (s, 0.84 × 3H), 2.11 (s, 0.48H), 2.10 (s, 0.48H), 1.33 (d, *J* = 6.7 Hz, 0.48H), 1.25(d, *J* = 6.7 Hz, 0.84 × 3H). MS (ESI) calculated mass for the parent C_12_H_18_O_8_ 290 [M], found 313 [M + Na].

*(3S,4R,5S,6S)-4-fluoro-6-methyltetrahydro-2H-pyran-2,3,5-triyl triacetate* **(22).** DAST (0.12 mL, 0.90 mmol) was slowly added to a solution of **21** (26 mg, 0.090 mmol) in anhydrous CH_2_Cl_2_ (1 mL) at −40 °C. The reaction was stirred at room temperature for 24 h. After being cooled down to −20 °C, MeOH (0.2 mL) was added and the solvent was removed under reduced pressure. The residue was diluted with CH_2_Cl_2_ (30 mL), washed with water, and dried over MgSO_4_. After the evaporation of solvents, the residue was purified using silica gel flash chromatography to afford product **22** as a colorless oil (14 mg, 54% yield). ^1^H NMR (400 MHz, CDCl_3_): δ 6.05 (d, 1H, *J* = 2.0 Hz), 5.19 (m, 1H), 4.87 (m, 1H), 4.38 (m, 1H), 4.26 (m, 1H), 2.13 (s, 3H), 2.12 (s, 3H), 2.11 (s, 3H), 1.36 (dd, 3H, *J* = 1.2 and 7.0 Hz). ^19^F NMR (376 MHz, CDCl_3_): δ −205.1. MS (ESI) calculated mass for the parent C_12_H_17_FO_7_ 292 [M], found 292 [M].

*(3S,4R,5S,6S)-4-fluoro-6-methyltetrahydro-2H-pyran-2,3,5-triol* **(23).** NaOMe (10 mg, 0.19 mmol) was added to a suspension of **22** (14 mg, 0.048 mmol) in dry MeOH (1.7 mL). The mixture was stirred at room temperature for 15 h. Then, the reaction mixture was neutralized with Dowex (H+) resin, filtrated, concentrated, and purified using silica gel flash column chromatography to afford **23** as a white solid (3.2 mg, 56% yield). ^1^H NMR (400 MHz, CD_3_OD): δ 4.93 (d, 0.41H, *J* = 1.2 Hz), 4.90 (d, 0.36H, *J* = 2.0 Hz), 4.22 (m, 0.36H), 4.28 (m, 0.36H), 4.10–4.03 (m, 0.82H), 3.97 (m, 0.41H), 3.80 (m, 0.36H), 3.51 (m, 0.41H), 3.38 (m, 0.36H), 1.30 (dd, 1.26H, *J* = 1.0 and 6.9 Hz); 1.25 (dd, 1.08H, *J* = 1.9 and 6.7 Hz); ^19^F NMR (376 MHz, CDCl_3_): δ −201.6, −206.1; HRMS (ESI) calculated mass for the parent C_6_H_11_FO_4_ 166.0641 [M], found 165.0565 [M − H].

*(3R,4S,5S,6S)-6-methyl-4-(((trifluoromethyl)sulfonyl)oxy)tetrahydro-2H-pyran-2,3,5-triyl triacetate* **(24).** Compound **24** (25 mg, 41% yield) was prepared using the same preparation procedure as compound **12**. ^1^H NMR (400 MHz, CDCl_3_): δ 5.94 (d, 1H, *J* = 2.0 Hz), 5.35 (m, 1H), 5.09 (dd, 1H), 4.89 (dd, 1H), 4.42 (dd, 1H), 2.16 (s, 3H), 2.15 (s, 3H), 2.12 (s, 3H), 1.46 (d, 3H); ^19^F NMR (376 MHz, CDCl_3_): δ −74.9. HRMS (ESI) calculated mass for the parent C_13_H_17_F_3_O_10_S 422.0495 [M], found 445.0377 [M + Na].

*(3S,5S,6R)-6-((trityloxy)methyl)tetrahydro-2H-pyran-2,3,4,5-tetrayl tetraacetate* **(26).** Triphenylmethyl chloride (3.4 g, 12.2 mmol) was added to L-Mannose **25** (2.00 g, 11.1 mmol) in anhydrous pyridine (10 mL). The mixture was stirred at room temperature for 15 h. A total of 6 mL of Ac_2_O was added afterwards and the solution was stirred for another 15 h. The mixture was poured into ice-cold water and extracted with EtOAc (3 × 100 mL). The combined organic layer was washed with brine and dried over Na_2_SO_4_. After the evaporation of solvents, the residue was purified using silica gel flash chromatography to afford product **26** as a white solid (5.84 g, 89%). ^1^H NMR (400 MHz, CDCl_3_): δ 7.46–7.22 (m, 15 H), 6.10 (s, 0.7H), 5.85 (s, 0.3H), 5.52 (m, 1H), 5.43–5.52 (m, 2H), 3.91 (m, 0.7H), 3.64 (m, 0.3H), 3.34 (m, 1H), 3.18 (0.3H), 3.07 (m, 0.7H), 2.24 (s, 2.1H), 2.23 (s, 0.9H), 2.17 (s, 2.1H), 2.14 (s, 0.9H), 2.00 (s, 2.1H), 1.98 (s, 0.9H), 1.76 (s, 0.9H), 1.75 (s, 2.1H). MS (ESI) calculated mass for the parent C_33_H_34_O_10_ 590 [M], found 613 [M + Na].

*(3S,5S,6R)-6-(hydroxymethyl)tetrahydro-2H-pyran-2,3,4,5-tetrayl tetraacetate* **(27).** 33% HBr in HOAc (1.6 mL) was added to the solution of compound **26** (4.60 g, 7.80 mmol) in glacial acetic acid (16 mL) at 10 °C. The mixture was stirred for 10 min. The formed triphenylmethyl bromide was immediately removed via filtration. The filtrate was diluted with cold water and extracted with EtOAc (3 × 100 mL). The combined organic layer was washed with water and brine and dried over Na_2_SO_4_. After the evaporation of solvents, the residue was purified using silica gel flash chromatography to afford product **27** as a white solid (2.23 g, 82%). ^1^H NMR (400 MHz, CDCl_3_): δ 6.09 (d, 0.67H, *J* = 1.6 Hz), 5.87 (d, 0.33H, *J* = 1.2 Hz), 5.49 (dd, 0.33H, *J* = 1.2 and 11.5 Hz), 5.40 (dd, 0.67H, *J* = 3.3 and 10.0 Hz), 5.33 (m, 0.67H), 5.27 (m, 1H), 5.17 (dd, 0.33H, *J* = 3.3 and 10.5 Hz), 3.85 (m, 0.67H), 3.73 (m, 1H), 3.66–3.58 (m, 1.33H), 2.21 (s, 0.99H), 2.17 (s, 2.01H), 2.16 (s, 2.01H), 2.10 (s, 0.99H), 2.08 (s, 2.01H), 2.04 (s, 0.99H), 2.02 (s, 2.01H), 2.01 (s, 0.99H). MS (ESI) calculated mass for the parent C_14_H_20_O_10_ 348 [M], found 371 [M + Na].

*(3S,4R,5R,6R)-6-(fluoromethyl)tetrahydro-2H-pyran-2,3,4,5-tetrayl tetraacetate* **(28).** Compound **28** (30 mg, 28%) was prepared using the same preparation procedure as compound **22**. ^1^H NMR (400 MHz, CDCl_3_): δ 6.11 (d, 0.57H, *J* = 2.0 Hz), 5.88 (d, 0.43H, *J* = 2.0 Hz), 5.49 (m, 0.43H), 5.38–5.35 (m, 2 × 0.57H), 5.31 (m, 0.43H), 5.26 (m, 0.57H), 5.15 (m, 0.43H), 4.56 (m, 1H), 4.44 (m, 1H), 4.02 (m, 0.57H), 3.80 (m, 0.43); 2.21 (s, 3 × 0.43 H), 2.17 (s, 3H), 2.16 (s, 3 × 0.57H) 2.11 (s, 3 × 0.43H), 2.07 (s, 3H), 2.01 (s, 3 × 0.57H); ^19^F NMR (376 MHz, CDCl_3_): δ −231.9, −232.4. MS (ESI) calculated mass for the parent C_14_H_19_FO_9_ 350 [M], found 350 [M].

*(3S,4R,5R,6R)-6-(fluoromethyl)tetrahydro-2H-pyran-2,3,4,5-tetraol* **(29).** Compound **29** (10 mg, 87%) was prepared using the same preparation procedure as compound **23**. ^1^H NMR (400 MHz, D_2_O/CD_3_OD): δ 5.18 (d, 0.6H, *J* = 2.0 Hz), 4.92 (d, 0.4H, *J* = 1.2 Hz), 4.78–4.57 (m, 2H), 3.93 (m, 1H), 3.87 (m, 1H), 3.77 (m, 1H), 3.68 (m, 1H). HRMS (ESI) calculated mass for the parent C_6_H_11_FO_5_ 182.0591 [M], found 181.0521 [M − H].

*3S,5S,6R)-6-((((trifluoromethyl)sulfonyl)oxy)methyl)tetrahydro-2H-pyran-2,3,4,5-tetrayl tetraacetate* **(30).** Trifluoromethanesulfonic anhydride (0.37 mL, 2.2 mmol) was added to a mixture of compound **27** (696 mg, 2.0 mmol) and pyridine (0.25 mL) in dichloromethane (20 mL) at −10 °C. After stirring for 2 h, water (50 mL) was added. The organic layer was separated and the aqueous layer was extracted with dichloromethane (3 × 50 mL). The organic layers were combined, washed with 10% H_2_SO_4_, sat. NaHCO_3_, and brine, and dried over MgSO_4_. After the evaporation of solvents, the residue was purified using silica gel flash chromatography to afford product **30** as a white solid (826 mg, 86%). ^1^H NMR (400 MHz, CDCl_3_): δ 6.12 (d, 0.62H, *J* = 2.0 Hz), 5.89 (d, 0.38H, *J* = 1.2 Hz), 5.49 (dd, 0.38H, *J* = 1.2 and 3.1 Hz), 5.39 (dd, 0.62H, *J* = 3.1 and 10.2 Hz), 5.33 (m, 0.62H), 5.30 (m, 0.38H), 5.26 (dd, 0.62H, *J* = 1.2 and 11.5 Hz), 5.16 (dd, 0.38H, *J* = 3.1 and 9.8 Hz), 4.58–4.54 (m, 2H), 4.14 (m, 0.62H), 3.92 (m, 0.38), 2.22 (s, 1.14H), 2.19 (s, 1.86H × 2), 2.12 (s, 1.14H), 2.10 (s, 1.86H), 2.05 (s, 1.14H), 2.03 (s, 1.86H), 2.02 (s, 1.14H). ^19^F NMR (376 MHz, CDCl_3_): δ −74.3, −74.4. HRMS (ESI) calculated mass for the parent C_15_H_19_F_3_O_12_S 480.0549 [M], found 503.0433 [M + Na].

### 3.3. Radiochemical Syntheses

All three tracers were prepared following the general procedure described below.

Radiosyntheses were performed on a GE Tracerlab FX-N2 synthesizer. The synthesis consisted of 7 reagent vials on the GE synthesizer. Vials 1–5 were used for the elution, the drying of fluorine-18, and the fluorination reaction. Vials 13–14 were used for the formulation of the purified intermediate. An inline K_2_CO_3_ cartridge was incorporated between valve 15 and the product vial. Specifically, the reagent vials contained the following: Vial 1: tetrabutylammonium bicarbonate solution (150 µL, 0.075 M), 50 µL water, and MeOH (1 mL); Vial 2: ACN (1 mL); Vial 3: triflate precursor (5 mg) in ACN (0.6 mL); Vial 4: water (1 mL); Vial 5: HPLC solvent (2.0 mL); Vial 13: EtOH (2 mL); Vial 14: water (6 mL); HPLC dilution flask: water (30 mL). The vial 11 inlet port was connected to the V15 right port to transfer the intermediate to reactor 2 (R2).

Typically, 7.4 GBq (200 mCi) [^18^F]fluoride in 2.5 mL of water was passed through a Chromafix PS-HCO_3_ cartridge, which was rinsed with 1 mL of acetonitrile. The retained [^18^F]fluoride was eluted from the cartridge into reactor 1 (R1) with the eluent in Vial 1 and dried under a N_2_/vacuum at 75 °C for 4 min. R1 was cooled to 50 °C, acetonitrile in Vial 2 was added and the activity was azeotropically dried at 55 °C for 3 min and at 95 °C for an additional 3 min under a N_2_/vacuum. The activity was further dried using a vacuum for 3 min. The [^18^F]fluoride drying cycle took about 20 min.

The triflate precursor solution in Vial 3 was added to the dried activity. The resulting solution was stirred at 70 °C for 20 min and cooled to 45 °C. The reaction mixture was diluted with 1.0 mL of water (Vial 4) and transferred into Tube 2. R1 was rinsed with HPLC mobile phase (Vial 5) and the solution was also transferred into Tube 2. The solution in Tube 2 was thoroughly mixed by bubbling N_2_ for 10 s and injected into the HPLC for purification. HPLC condition: Phenomenex Luna (2) C18 column, 250 × 10 mm, 5 µm. Mobile phase: 40% ACN in 0.1% TFA. Flow rate: 4 mL/min. The labeled intermediate was eluted for about 12–14 min which was collected in the dilution flask containing 30 mL of water and passed through an Oasis HLB plus cartridge (pre-conditioned with 5 mL of ethanol, 10 mL of air, and 10 mL of water). The trapped labeled intermediate was rinsed with 6 mL of water (Vial 14), eluted with 2 mL of absolute ethanol (Vial 13) through the inline K_2_CO_3_ cartridge to the vial with a stir bar, and then the mixture was stirred for 10 min. Saline pH 2.5 (2 mL) was added to the vial, the solution was passed through a preconditioned (18 mL ethanol and 30 mL water) FDG cartridge. The cartridge was washed with saline pH 2.5 (1 mL). The product was eluted with 4 mL of normal saline. The synthesis time was ~90 min.

### 3.4. Animals

All experimental procedures, including the handling and care of the animals, were approved by the Animal Care and Use Committee of the Clinical Center of the NIH and performed under relevant NIH policies. Healthy female CD-1 mice (aged 6–8 weeks, Charles River, Charleston, SC, USA) were used for the PET/CT imaging. All mice were housed with a 12-h light/dark cycle with free access to food and water.

### 3.5. PET/CT Imaging

The mice were first anesthetized with 3–4% isoflurane and the animals were kept warm using a heating pad during the scan.

A preclinical Inveon PET/CT scanner (Siemens Medical Solutions, Malvern, PA, USA) was used. Each animal was first secured onto the scanner bed and placed symmetrically within the center FOV. A CT scan was then performed for attenuation correction and anatomic guidance when placing the volumes of interest for the calculation of radioactivity concentrations (VOIs). Immediately after the PET acquisition was started, the tracer was injected via the tail vein (~9 MBq) as a bolus (*n* = 4), followed by a quick saline flush (total ~200 μL). Dynamic PET images were acquired over a period of 60 min in list mode. The emission sinograms were corrected for scatter, ^18^F-decay, random, and dead time. The resulting histograms were then reconstructed by applying Fourier rebinning and the 3D ordered subject expectation maximization algorithm (OSEM-3D). The images were analyzed with pMOD 3.2. For dynamic PET imaging with 3-deoxy-3-[^18^F]-fluoro-L-rhamnose, a Mediso PET/CT scanner was used. The imaging methodology and the dose administered did not differ from the previous scans conducted on the Inveon imager. The images were analyzed using Fusion software (Mediso Ltd., Budapest, Hungary).

## 4. Conclusions

Three fluorine-18-labeled L-rhamnose derivatives have been developed for PET imaging studies with the ultimate goal of using those compounds to image various infectious diseases. Using triflate precursors, these new radioligands were prepared with medium to high RCY. The radiolabeling methods were successfully automated with the intention of large-scale production. All three derivatives were used for PET/CT imaging in mice. Of these, 3-deoxy-3-[^18^F]fluoro-L-rhamnose and 6-[^18^F]fluoro-L-rhamnose showed rapid defluorination after in vivo injection (Figure 6). On the other hand, 2-deoxy-2-[^18^F]fluoro-L-rhamnose was found to be very stable upon injection in healthy mice. This ligand rapidly cleared and there was minimal uptake in various organs indicating lack of uptake and/or metabolism by mammalian cells. There was also no liver metabolism detected and excretion was completely via the renal route (Figure 5). Furthermore, unlike FDG, there was no discernable accumulation of the ligand in the myocardial tissues. We are only describing uptake of radiolabeled L-Rhamnose in healthy animals. We are currently finalizing in vitro and in vivo uptake results in different animal models of infection in order to establish the usefulness of radiolabeled Rhamnose in detecting infection in vivo. The results will be published separately.

## Figures and Tables

**Table 1:** Stability of tracers at 37 °C in whole human serum.

Tracer	% Intact			
	1 h	2 h	3 h	4 h
**5**	98	98	98	98
**6**	97	82	78	70
**7**	98	98	97	92

## Data Availability

Data is contained within the article and Supplementary Materials.

## Supplementary Materials

The following supporting information can be downloaded at: https://www.mdpi.com/article/10.3390/molecules28093773/s1.
