# Supplementary material for: Synthesis and Evaluation of Fluorine-18-Labeled L-Rhamnose Derivatives
Source: Molecules. 2023 Apr 27;28(9):3773. doi: 10.3390/molecules28093773 (PMC10180268; doi:10.3390/molecules28093773)
Supplement: Supplementary file 1 [file molecules-28-03773-s001.zip › molecules-2291330-supplementary.pdf]

Synthesis and Evaluation of Fluorine-18 labeled L-Rhamnose Derivatives

Xiang Zhang[1] *, Falguni Basuli[1], Zhen-Dan Shi[1], Swati Shah[2], Jianfeng Shi[1], Amelia Mitchell[2], Jianhao Lai[2], Zeping Wang[2], Dima A. Hammoud[2 †], Rolf E. Swenson[1 †]

[1] Chemistry and Synthesis Center, National Heart, Lung, and Blood Institute, National Institutes of Health, Rockville, MD, 20850, USA

[2] Center for Infectious Disease Imaging, Radiology and Imaging Sciences, Clinical Center, National Institutes of Health, Bethesda, MD, 20892, USA

* Correspondence: xiang.zhang2@nih.gov

† These authors contributed equally to this work.

Figure S1. Radio-TLC chromatogram of A) Compound **5;** B) Compound **6**; C) Compound **7** in whole human serum at 37 °C from 0–4 h. TLC developing solvent: 25% methanol in dichloromethane.

A)

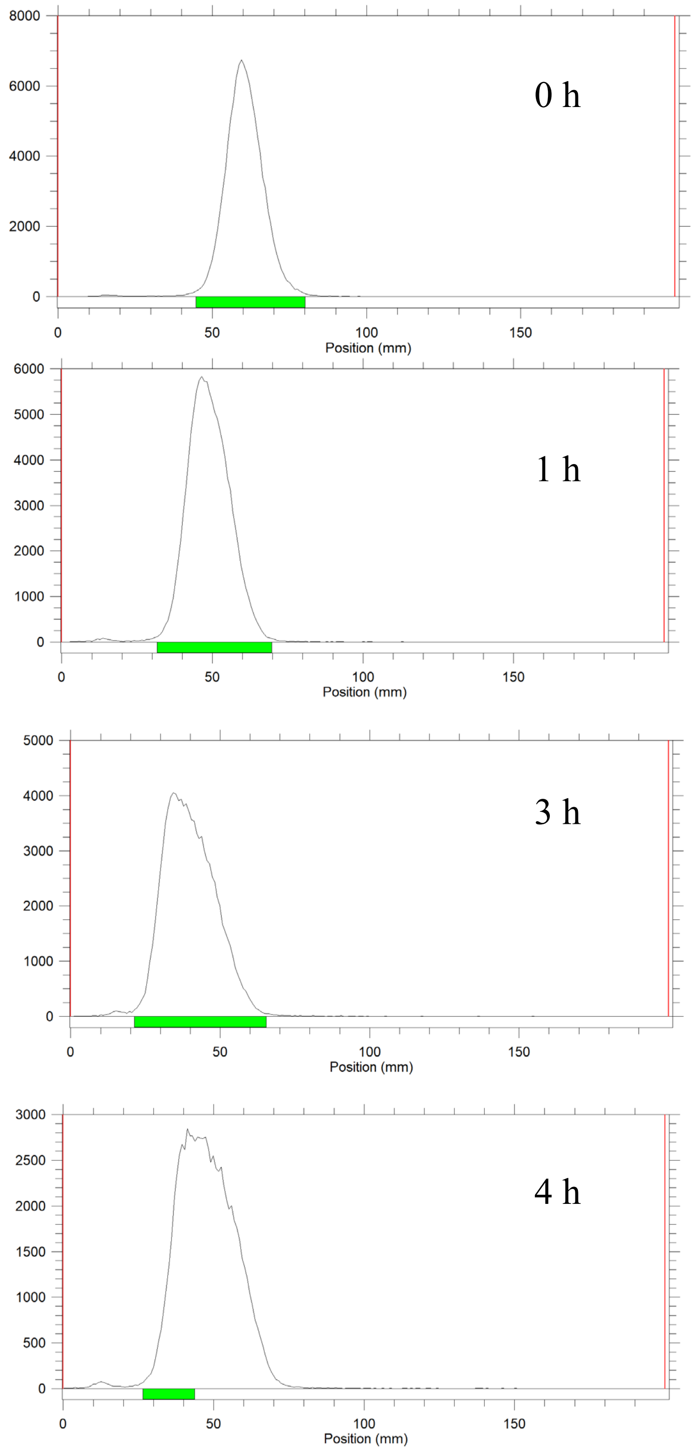

B)

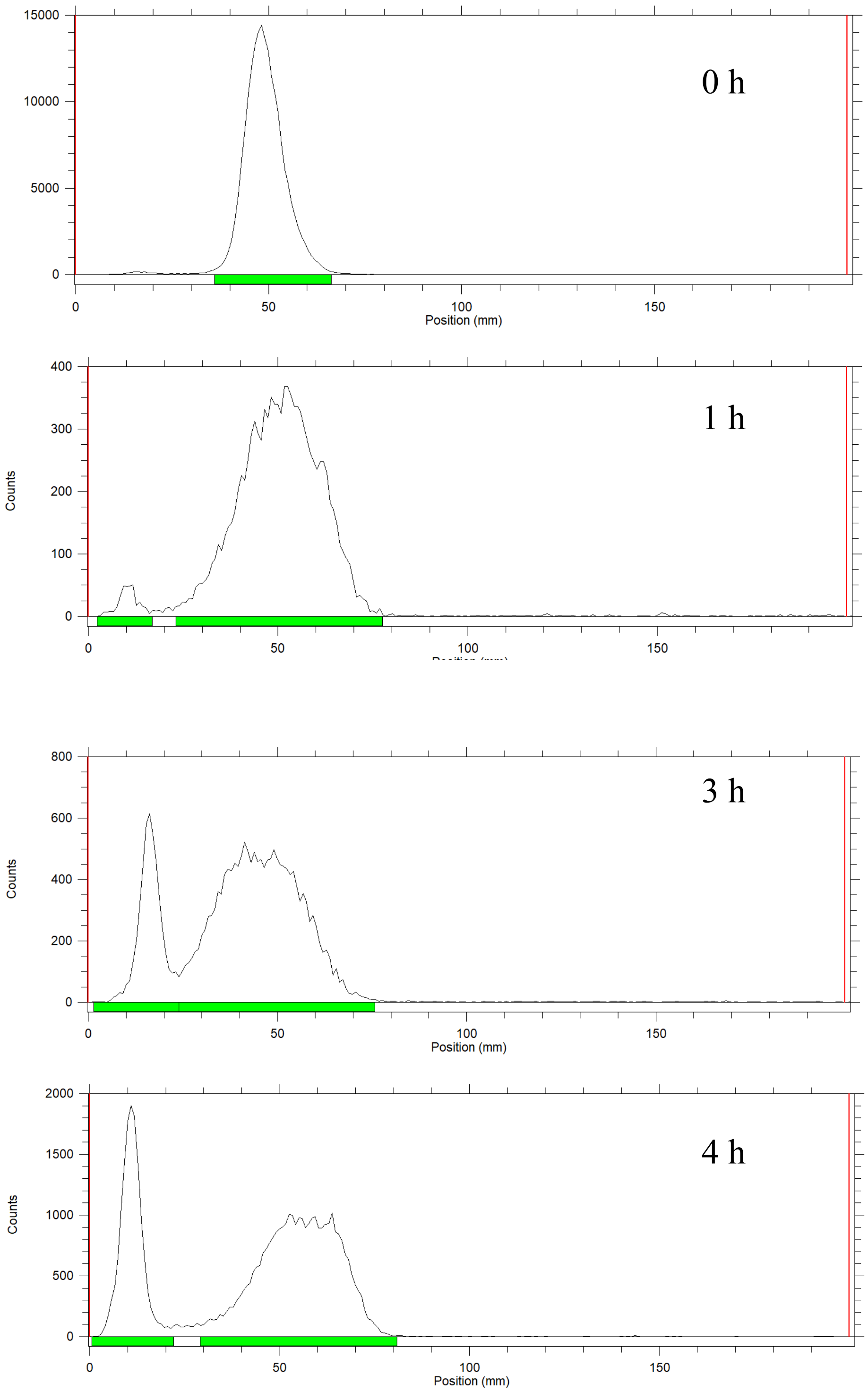
0 h
1 h
3 h
4 h
Counts
Position (mm)
15000
10000
5000
0
400
300
200
100
800
600
2000
1500
1000
500
50
100
150

c)

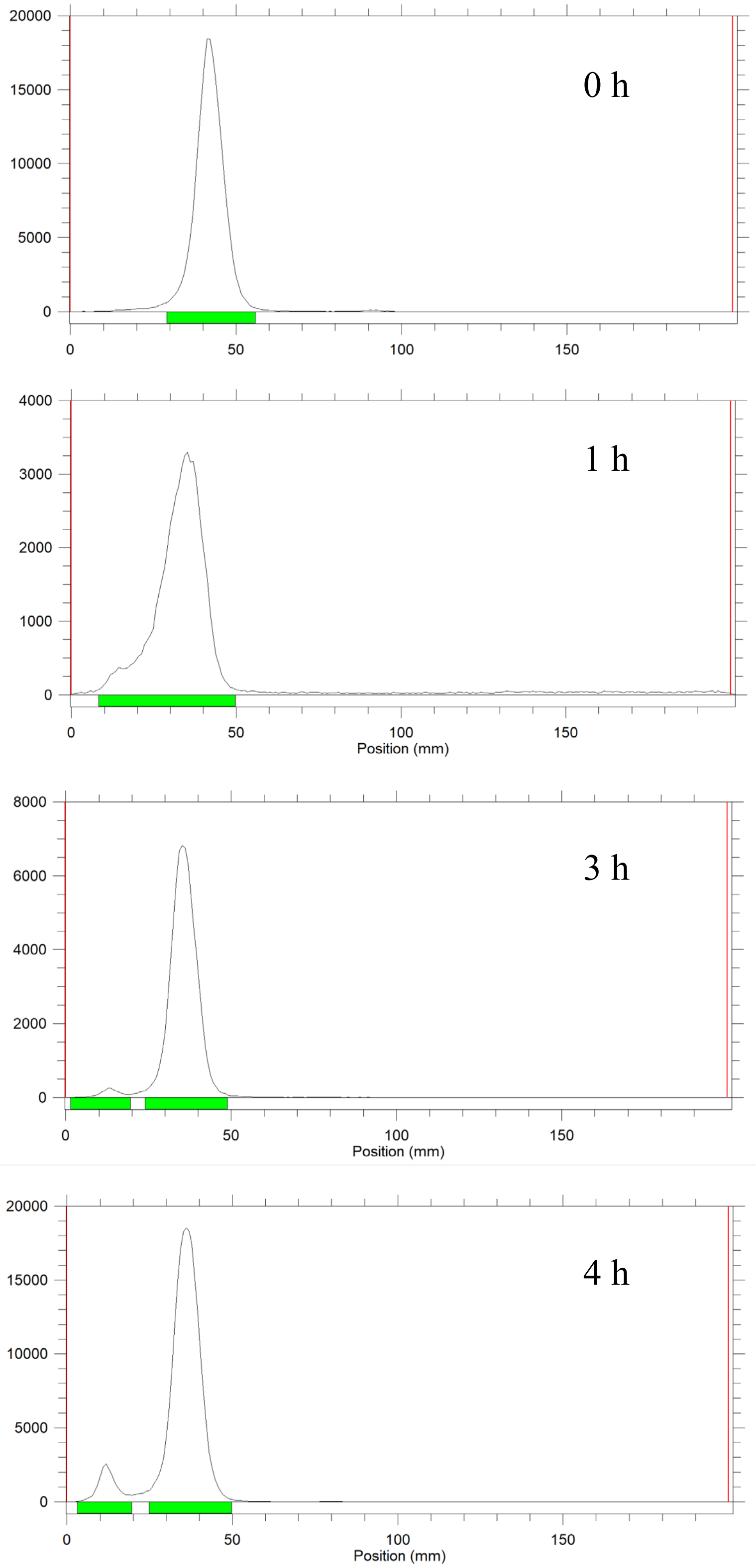
0 h
1 h
3 h
4 h
20000
15000
10000
5000
4000
3000
2000
1000
8000
6000
0
50
100
150
Position (mm)
